# Supplementary figures and images for: Historical contingency in parasite community assembly: Community divergence results from early host exposure to symbionts and ecological drift
Source: PLoS One. 2023 May 16;18(5):e0285129. doi: 10.1371/journal.pone.0285129 (PMC10187916; doi:10.1371/journal.pone.0285129)

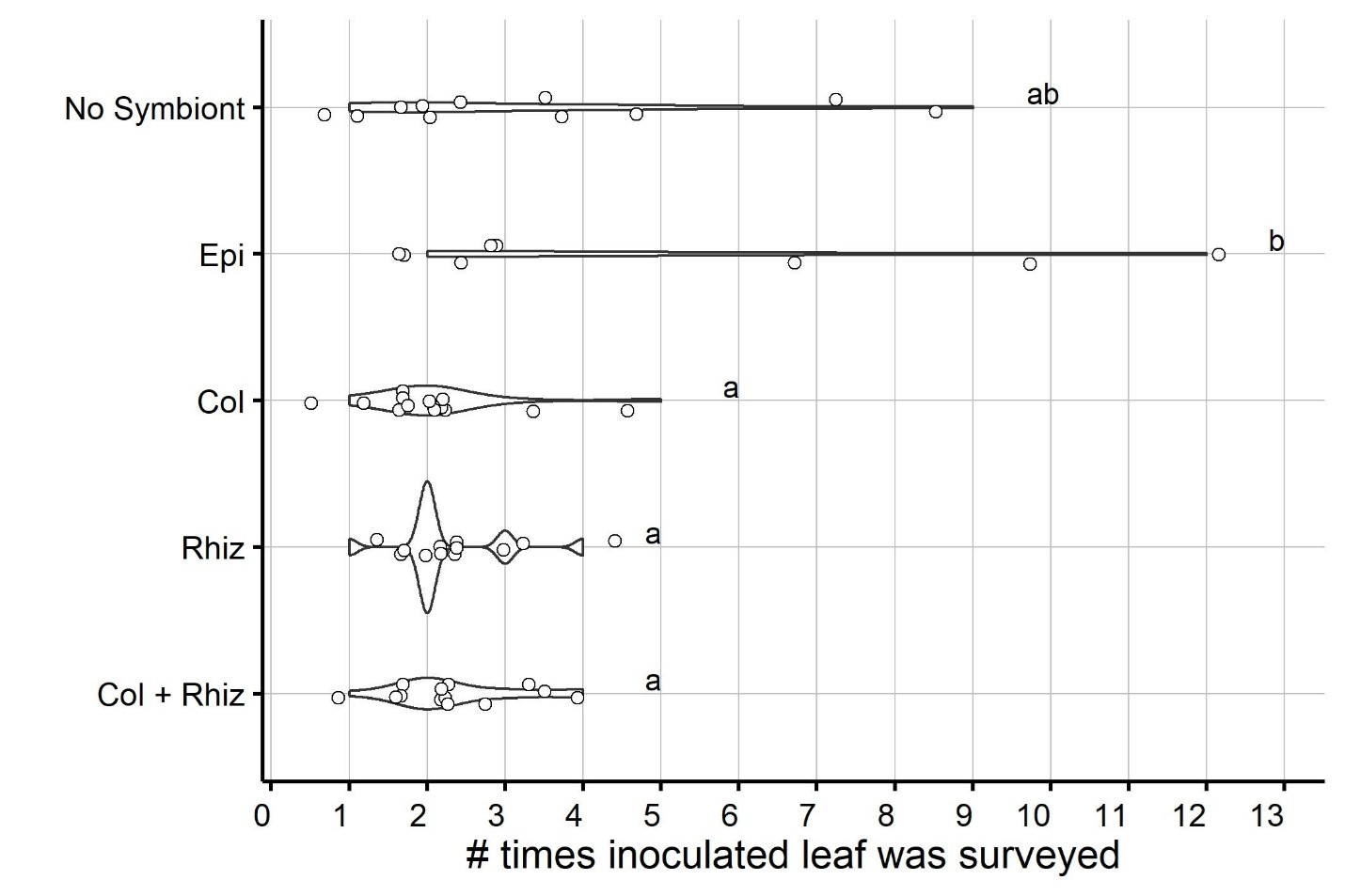

Supplement: S1 Fig — Each point indicates an individual leaf that received an inoculation (or mock inoculation) treatment and how many times that inoculated leaf was surveyed in the field. Inoculations were implemented on the oldest living leaf prior to being outplanted in the field. Note that points are jittered to show the distribution of the raw data. Letter denote grouping based on Tukey HSD post-hoc comparisons. (TIF) [file pone.0285129.s001.tif]

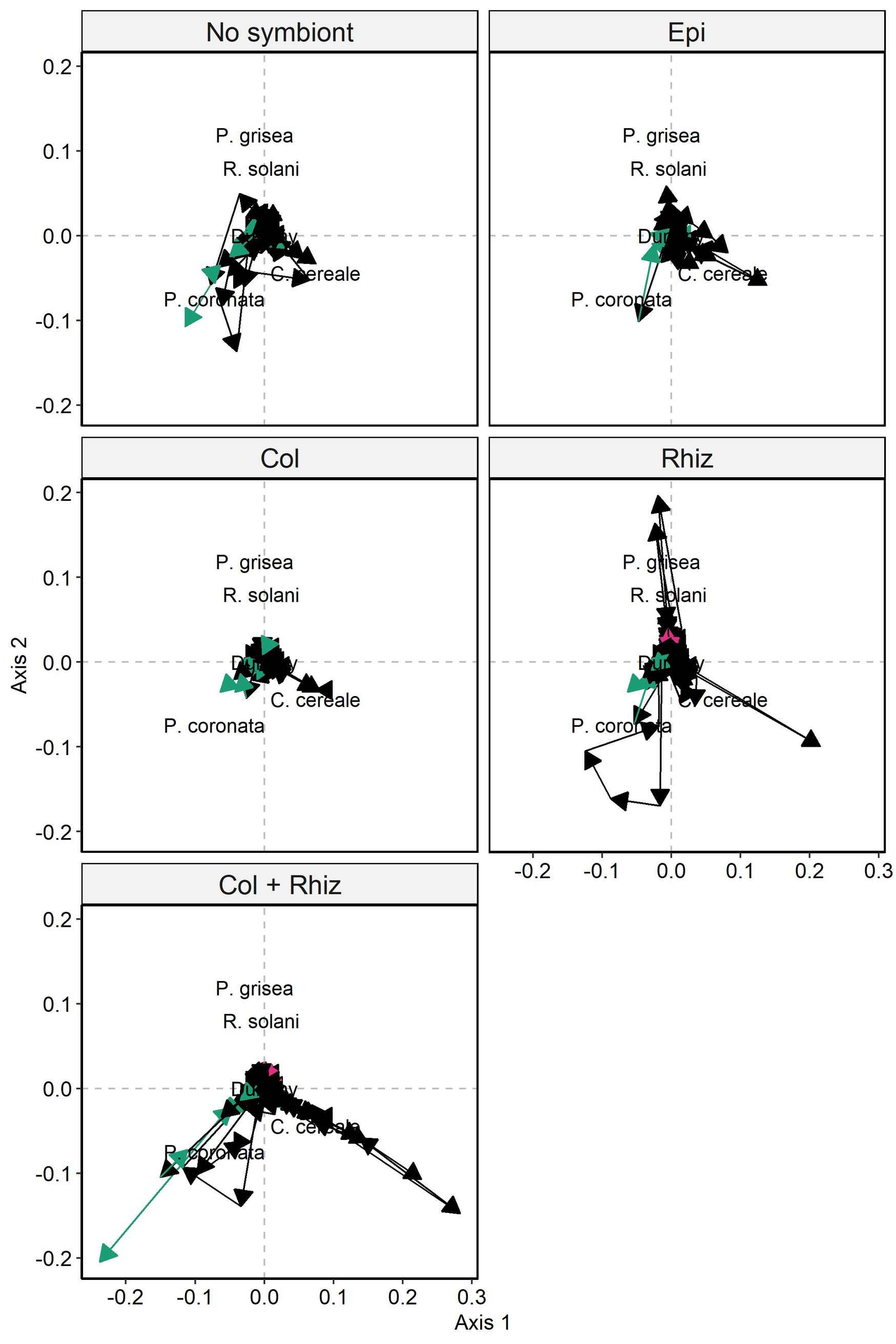

Supplement: S2 Fig — Each panel depicts the parasite community trajectory of each host individual within an inoculation treatment group. Direction of arrows indicate path of each host’s parasite community trajectory. Green arrows represent the ending community state for each host. (TIF) [file pone.0285129.s002.tif]

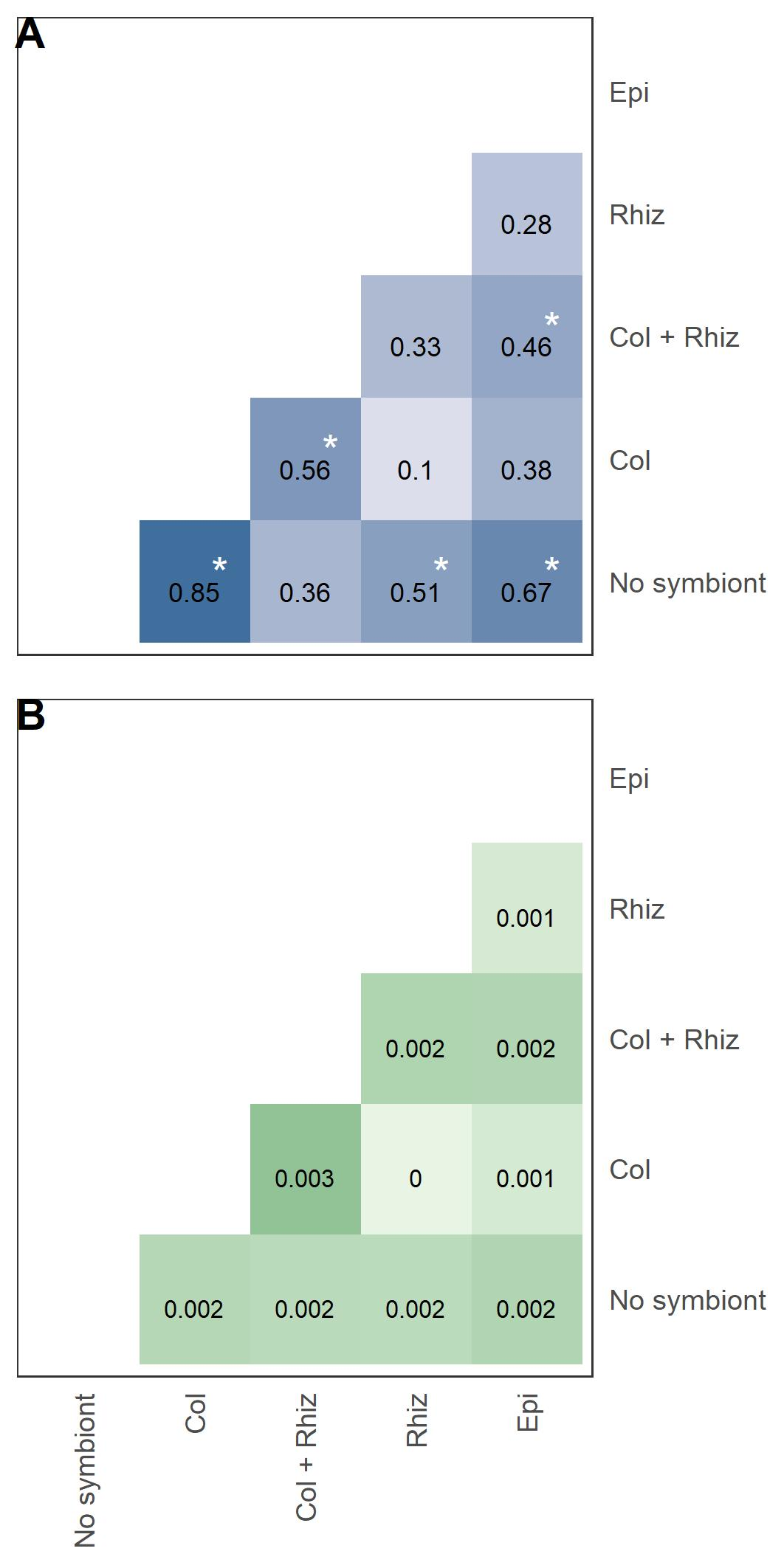

Supplement: S3 Fig — Parasite community trajectories showed A) trends of divergence between most inoculation groups, and B) the magnitude of divergence between communities was greatest for parasite communities from hosts co-inoculated with both parasite species, C. cereale and R. solani. Panel A shows the results of pairwise Mann-Kendell trend tests, which test for trends of convergence and divergence between communities. Within cells are Tau values, which indicate whether trajectories show signals of convergence (Tau < 0) or divergence (Tau > 0); the fill of the cells correspond to Tau values, where white cells are 0 and darker blue cells are closer to 1. Panel B shows the values of pairwise sens slopes, which measures the magnitude of divergence. Within cells are the sens slope values, where darker green cells indicate a greater magnitude of divergence between community trajectories. Asterisks in panel A denote significant (p<0.05) trends of convergence or divergence, and these p-values also correspond to sens slopes in panel B. (TIF) [file pone.0285129.s003.tif]

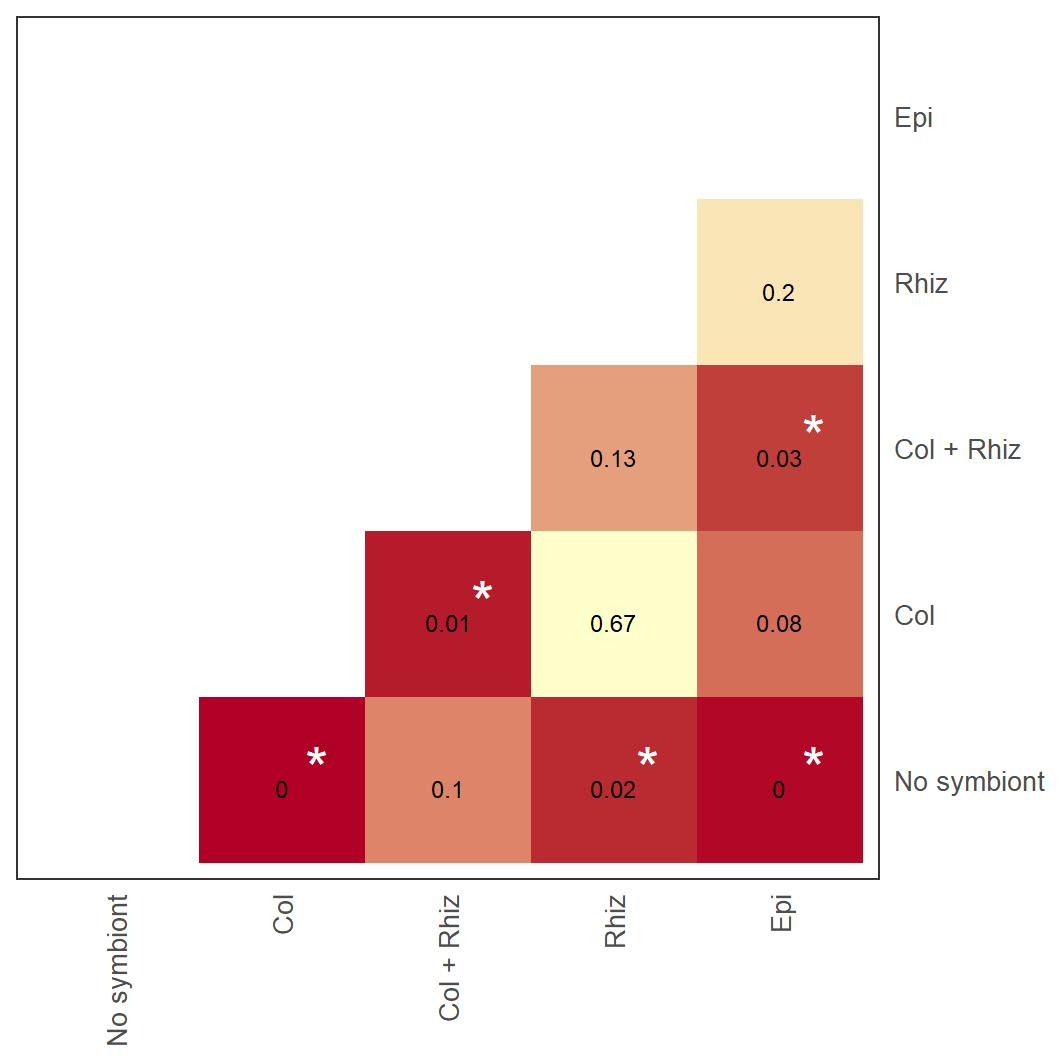

Supplement: S4 Fig — Asterisks denote significant (p<0.05) trends and sens slopes. Within cells are p-values and the fill of the cells correspond to p-values, where yellow cells are closer to p = 1 (non-significant trend) and darker red cells are closer to p = 0. (TIF) [file pone.0285129.s004.tif]

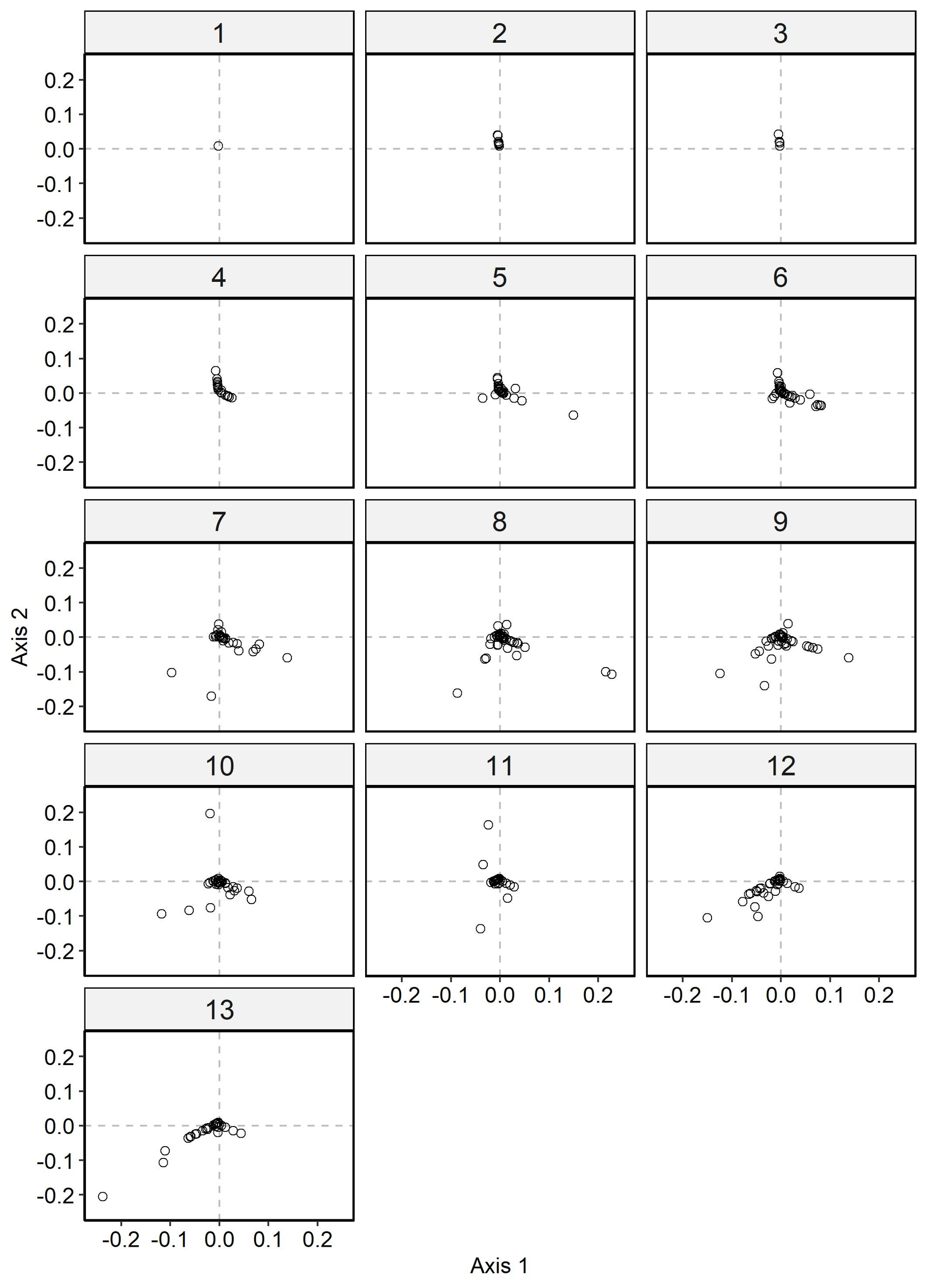

Supplement: S5 Fig — Each panel shows the spread of parasite community structure across all plants for a given sampling event. On the first survey event (panel 1) communities were identical and variation among communities increase throughout assembly. (TIF) [file pone.0285129.s005.tif]
