# Supplementary material for: Historical contingency in parasite community assembly: Community divergence results from early host exposure to symbionts and ecological drift
Source: PLoS One. 2023 May 16;18(5):e0285129. doi: 10.1371/journal.pone.0285129 (PMC10187916; doi:10.1371/journal.pone.0285129)
Supplement: S1 Table — The # of leaves indicate counts of all leaves that were at any point infected with the target parasite, % prev. is the percent of surveyed leaves that were at any point infected with the target parasite, severity is the average severity across the entire experiment, and total leaves are the number of leaves that were at any point infected by any parasite. (DOCX) [file pone.0285129.s006.docx]

| Inoculation treatment | *Rhizoctonia* infections | | |  | *Colletotrichum* infections | | |  | total leaves |
| --- | --- | --- | --- | --- | --- | --- | --- | --- | --- |
|  | # leaves | % prev. | severity |  | # leaves | % prev. | severity |  |  |
| No symbiont | 11 | 33 | 0.102 |  | 20 | 61 | 0.372 |  | 33 |
| Epi | 7 | 18 | 0.256 |  | 20 | 48 | 0.651 |  | 42 |
| Col | 5 | 17 | 0.027 |  | 17 | 57 | 0.331 |  | 30 |
| Rhiz | 11 | 36 | 0.577 |  | 17 | 55 | 0.456 |  | 31 |
| Col + Rhiz | 8 | 20 | 0.040 |  | 29 | 72 | 1.111 |  | 40 |
