# Supplementary material for: Historical contingency in parasite community assembly: Community divergence results from early host exposure to symbionts and ecological drift
Source: PLoS One. 2023 May 16;18(5):e0285129. doi: 10.1371/journal.pone.0285129 (PMC10187916; doi:10.1371/journal.pone.0285129)
Supplement: Attachment — Submitted filename: Letter_R2.docx [file pone.0285129.s008.docx]

Dear Editor,

Thank you for the opportunity to submit a revised version of our manuscript titled “Historical contingency in parasite community assembly: community divergence results from early host exposure to symbionts and ecological drift.” We thank the reviewers for their comments and have revised the manuscript in response to Reviewer #2.

We have provided our response to the comments with line references below in ***bold italics***.

We feel these changes have improved the quality of our manuscript, and we hope that you will now find this work suitable for *PLOS One*. Thank you for your efforts in the publication of our research.

Sincerely,

Rita Grunberg

**Review Comments to the Author**

Reviewer #2: As I indicated in my first review, this study concerns an interesting and little studied topic in community ecology. The combination of experimental and field setups is appropriate and I believe provides interesting insight into the assembly of pathogen-mutualist communities in tall fescue.

I am satisfied with the answers and corrections done by the authors. I would highlight in particular the addition of an assessment of the contribution to the field and a discussion of applicability to other host-parasite/mutualist systems as elements that add value to the study. Thus, I have no reservation in recommending the article for publication in PLOS One.

There are some minor issues that the authors should consider:

1. Table S1 contains information that is highly relevant for the reader to grasp the structure of the experimental design. I would move it to the main text.

***Author: We moved Table S1 to the main text (see lines 177). We agree it will help the reader understand the experimental design.***

2. Here and there (e.g., 282-283), C. cereale is written with an additional blank space.

***Author: Fixed.***

3. 301 – Replace “distance” with “dissimilarity”. The Bray Curtis metric is a dissimilarity, not a distance (see e.g. Roberts 2017, https://doi.org/10.1111/2041-210X.12739, for details).

***Author: Fixed.***

4. 482 – “As predicted by theory”. Please provide a reference.

***Author: We added the corresponding reference; Seabloom et al (2015). The community ecology of pathogens: Coinfection, coexistence and community composition. Ecology Letters, 18(4), 401–415.***
